# Unusual Noncommunicating Isolated Enteric Duplication Cyst in Adults

**DOI:** 10.1155/2011/323919

**Published:** 2011-05-05

**Authors:** Metehan Gümüş, Murat Kapan, Hatice Gümüş, Akin Önder, Sadullah Girgin

**Affiliations:** ^1^Department of General Surgery, Medical Faculty, Dicle University, 21280 Diyarbakir, Turkey; ^2^Department of Radiology, Medical Faculty, Dicle University, 21280 Diyarbakir, Turkey

## Abstract

Duplication cysts are rare gastrointestinal congenital abnormalities and can occur anywhere within the gastrointestinal tract. Duplication cysts are firmly attached to or share the wall of the alimentary tract and have a common blood supply with the adjacent segment of the bowel. Completely isolated duplication cysts are an extremely rare variety of gastrointestinal duplications with their own exclusive blood supply, and they do not communicate with the intestine. These cysts are usually diagnosed during early childhood, and very rarely detected in adults, mostly incidentally, due to a lack of symptoms. A 28-year-old male was admitted to our hospital with a chief complaint of lower abdominal pain and distention and a palpable mass for 1 month. Based upon computed tomography and sonographic findings, a small bowel duplication cyst was tentatively diagnosed. The cyst had no connection to the gastrointestinal tract. Herein we report the case of a noncommunicating isolated ileal duplication cyst in an adult. Resection of the cyst was performed safely without requiring bowel resection.

## 1. Introduction

Enteric duplication cysts are uncommon congenital abnormalities that originate anywhere along the alimentary tract from the tongue to the anus. A small bowel duplication cyst is the most common type of enteric duplication cyst, and the ileum is the most common location [1, 2]. The prevalence of duplication cysts is 2-fold higher in women, and they show no familial aggregation [3]. Diagnosis is made in more than half of the cases during early childhood because duplication cysts tend to be symptomatic in this age group. Conversely, these cysts are usually asymptomatic during adulthood, and the diagnosis is mostly incidental. In almost half of the cases, duplication cysts are associated with other malformations, mainly located in the esophagus and vertebrae. Complications, such as bleeding, fistulization, and even malignant degeneration, are associated with duplication cysts [4]. Completely isolated duplication cysts are an extremely rare variety of gastrointestinal duplications with their own blood supply, and they do not communicate with the normal bowel segment [5–7]. Herein we report an adult male with a noncommunicating ileal duplication cyst.

## 2. Case Report

**Figure 1:**
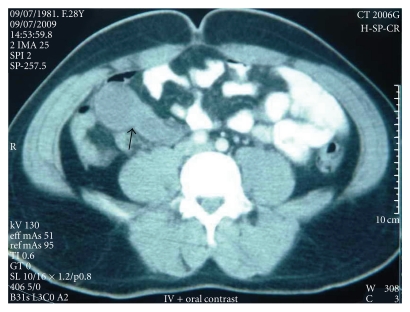
Lobulated cystic mass with slightly enhancing smooth walls in the right lower peritoneal cavity adjacent to the small bowel loops. None of the orally administered opaque material was in the cystic cavity.

**Figure 2:**
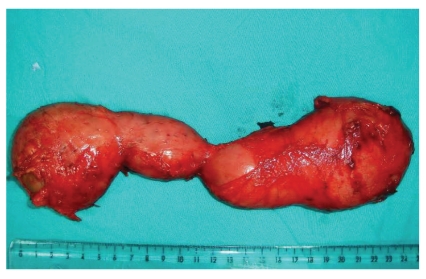
Resected ileal duplication cyst. Intact mucosal layer seen in left pole of cyst.

A 28-year-old male was admitted to our hospital with a chief complaint of lower abdominal pain and distention, and a palpable mass which had been more evident within last month. On physical examination, there was tenderness and a semimobile mass in the right lower abdomen. In abdominal contrast-enhanced computed tomography, two lobulated cystic masses which were in association with one another, with slightly enhancing smooth walls (110 × 35 mm and 95 × 45 mm in diameter), were demonstrated in the right lower peritoneal cavity which is adjacent to the small bowel loops (Figure 1). None of the orally administered opaque material was in the cystic cavity. On sonography, they abutted the adjacent small bowel and had an anechoic cystic center and a smooth echogenic peripheral wall. At laparotomy, there was a large 25 × 5-6 cm tubular fluid-filled cystic bowel-like mass extending from 20 cm proximal to the ileocecal junction toward the root of mesentery, which was blind-ended (Figure 2). The entire cyst was excised without disturbing the normal bowel or mesenteric anatomy. There was no relationship between the cystic and ileal lumens. After removal of the cyst, there was a small defect on the serosal layer, but the mucosal layer was intact. The histopathologic examination of the tissue revealed an enteric duplication cyst. No postoperative complications occurred, and the patient remained well in followup.

## 3. Discussion

Duplication cysts were first described by Wendel in 1911, and few cases have been reported since that time [3]. Enteric duplication cysts are hollow, epithelium-lined, cystic, spherical, or tubular structures that are basically attached to the wall of the gastrointestinal tract (often sharing the serosa) and supplied by common mesenteric blood vessels [7]. Enteric duplication cysts usually share a common wall with the normal intestine and have a common blood supply, so it is mandatory to remove the adjacent bowel segment along with duplication cyst [5, 7]. In our case, the isolated duplication cyst rested on the mesentery with a separate vascular pedicle and no luminal communication with the adjacent alimentary segments [7]. Intestinal duplications are usually symptomatic and present within the first year of life with intestinal obstruction or a palpable mass. Adults may experience similar symptoms, with acute presentations attributed to recent hemorrhage from ulceration or malignant transformation within the duplication [4, 8]. Unrecognized, asymptomatic cysts may be the site of adenocarcinoma during adult life. The enteric duplication cyst can be associated with malrotation [7]. In half of the cases, there are associated malformations, the most frequent of these being esophageal duplications, followed by vertebral abnormalities [3]. No malrotation and malformations as esophageal duplications and vertebral abnormalities were seen in our case. Imaging techniques, such as computed tomography (CT) and magnetic resonance imaging, do not accurately diagnose these lesions and perform rather poorly in terms of lesion characterization. On the other hand, recent reports suggest that endoscopic ultrasonography (EUS) may play a major role in the diagnosis of this disease, showing higher accuracy rates versus traditional imaging techniques [3]. Ultrasonography and CT scanning may confirm the cystic nature of duplication cysts. Ultrasonography shows a hypoechoic mass with strong posterior wall echoes and good through transmission due to clear fluid content or an echogenic mass due to hemorrhage and inspissated material within the duplication. In our case, the cyst had a clear fluid content. If the typical inner echogenic mucosal and outer hypoechoic muscle layers are seen on ultrasonography, the diagnosis of a duplication cyst can be established [4, 9]. Duplication cysts can be recognized on CT as smoothly rounded, fluid-filled cysts, or tubular structures with thin, slightly enhancing walls in or adjacent to the wall of part of the alimentary tract [4]. In our case, based upon CT and sonographic findings, a small bowel duplication cyst was tentatively diagnosed. The differential diagnosis includes all cystic intra-abdominal masses, such as mesenteric and omental cysts, pancreatic pseudocysts, and ovarian cysts [4, 8]. There is no worldwide accepted therapeutic algorithm for duplication cysts. Surgery is recommended for symptomatic cases or when a complication arises. However, to date there is no consensus on the management of asymptomatic cases [3]. In our case, the patient had a symptomatic duplication cyst and underwent surgical excision.

## 4. Conclusion

Duplication cysts should be considered in the differential diagnosis of abdominal cystic lesions. In the case of completely isolated duplication cysts, resection can be accomplished safely without requiring bowel resection, as demonstrated in our case.

##  Conflict of Interests

All authors have no financial interests relating to the manuscript.
